# Effectiveness of a Nature Sports Program on Burnout Among Nursing Students: A Clinical Trial

**DOI:** 10.3390/healthcare13192510

**Published:** 2025-10-02

**Authors:** Inmaculada Pérez-Conde, Nora Suleiman-Martos, María José Membrive-Jiménez, María Dolores Lazo-Caparros, Sofía García-Oliva, Guillermo A. Cañadas-De la Fuente, Jose Luis Gómez-Urquiza

**Affiliations:** 1 Nursing Department, Ceuta Faculty of Health Science, University of Granada, 51005 Ceuta, Spain; inmaconde@ugr.es (I.P.-C.); mjmembrive@ugr.es (M.J.M.-J.); sofiagarcia@ugr.es (S.G.-O.); jlgurquiza@ugr.es (J.L.G.-U.); 2 Faculty of Health Sciences, University of Granada, Av. Ilustración 60, 18016 Granada, Spain; gacf@ugr.es; 3 Brain, Mind and Behaviour Research Center (CIMCYC), University of Granada, Campus Universitario de Cartuja s/n, 18011 Granada, Spain; 4 Ceuta University Hospital, INGESA, 51003 Ceuta, Spain; mdlazo@correo.ugr.es

**Keywords:** sport, academic burnout, nursing, university students, prevention, mental health

## Abstract

**Background/Objectives**: Academic burnout is an emerging problem among nursing students, characterized by emotional exhaustion, cynicism, and reduced academic efficacy. Sports interventions have been shown to have a positive effect on nurses as a preventive strategy against burnout. The aim of this study was to evaluate the effect of a nature sports program on the levels of academic burnout in nursing students. **Methods**: A randomized clinical trial was performed. The intervention was a 12-week nature exercise program with two sessions each week. The main dependent variables were burnout (measured using the Maslach Burnout Inventory—Student Survey), stress (measured using the Perceived Stress Scale), and anxiety and depression (measured using the Hospital Anxiety and Depression Scale). The post-intervention sample size was n = 58 in the control group and n = 48 in the intervention group. **Results**: After the intervention, significant differences were found in respect of emotional exhaustion (*p* < 0.001; Cohen’s D: 0.483), stress (*p* < 0.05; Cohen’s D: 0.456), and mean steps per day (*p* < 0.001; Cohen’s D: −1.09), with the mean values being reduced in the intervention group by around three points in emotional exhaustion and stress; the intervention group also achieved a higher mean number of daily steps compared to the control group. **Conclusions**: A nature sports program could help to reduce emotional exhaustion and stress, and increase the number of steps per day.

## 1. Introduction

The term “burnout” comes from the English verb “burn,” which can mean “to consume oneself”, and the adverb “out”, which means outside, extinguished, finished. From these two terms comes the word “burnout”, which means “to be burned out” [1].

Before burnout was discussed scientifically, in the field of psychiatry, specialists Schwartz and Will published a description of a nurse affected by this syndrome in the journal Psychiatry in 1953. They described the case of Miss Jones, who worked in a psychiatric hospital and showed symptoms of exhaustion, irritability, demotivation, and indifference towards patients and colleagues. “Her depressed mood made her irritable, she felt exhausted and was insensitive and indifferent, particularly towards patients. Now Miss Jones only sees the negative side of her work and avoids social contact with patients and colleagues” [2].

Burnout syndrome was first discussed in 1969, when Bradley [3] described it as a psychological disorder. A few years later, in 1974, Freudemberg [4] studied the behavior of workers at an addiction center, finding that they were increasingly tired, dissatisfied, and showing less interest in their work. Over time, this caused some of these people to develop symptoms of depression, anxiety, and stress.

In 1986, Cristina Maslach [5] and her team devised a system called the Maslach Burnout Inventory (MBI) to assess burnout syndrome. This test-based system has been modified over the years. The current MBI includes three dimensions of burnout: emotional exhaustion (EE), depersonalization (D), and reduced personal accomplishment (PA). The Likert-type test consists of seven possible responses rated from 0 to 6. This questionnaire has been validated, adapted, and translated into different languages [6]. In Spain, it was adapted by Seisdedos [7] in 1997.

Following the parameters of the World Health Organization (WHO), which defines health in its constitution as “a state of complete physical, mental, and social well-being and not merely the absence of disease or infirmity” (1946) [8], it can be inferred from this definition that protecting the mental health of workers is essential to achieving optimal health.

The WHO has developed the “Comprehensive Mental Health Action Plan 2013–2030,” whose purpose is “to promote mental well-being, prevent mental disorders, provide care, improve recovery, promote human rights, and reduce mortality, morbidity, and disability among people with mental disorders” [9].

Burnout goes beyond “being burned out” at work; it is a problem that, due to its characteristics, can have a severe impact on people’s physical and mental health [10].

Burnout is pervasive across occupational professionals; however, nurses remain a critical focus given their statistically elevated risk, system-sustaining responsibilities amid high time-pressure, enduring expectations of empathy, and the consequential impacts on patient safety and overall health-system performance [11], which in turn creates real problems at the organizational level and in terms of worker protection [12]. This is a significant issue, as workplaces are affected by absenteeism, reduced staffing levels, increased waiting times, and a decline in the quality of care [13].

Among the groups that are susceptible to burnout are university students, with nursing students experiencing high levels of stress and burnout during their training [14,15]. Some studies indicate that one way for university students to reduce the symptoms of burnout is to maintain healthy lifestyle habits, including proper nutrition, meditation, good sleep hygiene, and physical activity [16,17]. Physical activity has been shown to have a positive impact in reducing burnout among university students. Evidence indicates that exercise performed in natural settings yields greater acute improvements in psychological outcomes than comparable indoor activity, producing immediate reductions in anxiety and stress levels relative to an indoor exercise program, whereas enjoyment does not differ significantly between environments [17].

Thus, it is important to assess whether these effects are also observed in nursing students and whether engaging in exercise outdoors in nature can have an even greater impact. It is hypothesized that nursing students who participate in a structured 12-week nature-based sports program will exhibit significantly lower levels of burnout, anxiety, depression, and stress, and engage in more physical activity, compared to those in the control group.

The aim of this study was to analyze the effectiveness of a physical activity program in nature on burnout in nursing students.

## 2. Materials and Methods

A randomized clinical trial was conducted. The writing was undertaken following the CONSORT checklist (Supplementary Material) [18]. This study was registered in OSF in August of 2025 (osf.io/93ndg).

### 2.1. Participants

Nursing students in their second year at the University of Granada were included in this study. These students had not yet begun their clinical training and continued to attend the campus for theoretical lessons. Students diagnosed with anxiety disorders or depression were excluded to avoid potential confounding variables. Baseline data were collected in the first week of March and post-intervention data after 12 weeks of intervention were collected in May 2025.

### 2.2. Sample Size

The sample size was calculated using G*Power 3.1 software, using a two-tailed Student’s *t*-test for comparison of means. A moderate effect size (d = 0.5), a significance level of 0.05, and 80% statistical power (1 − β = 0.80) were assumed, with equal allocation between groups (1:1). Under these parameters, a total of 128 participants (64 per group) was estimated to be required to detect statistically significant differences in burnout levels based on sports practice. All the students from the second course (n = 132) were invited to participate in this study and randomized.

### 2.3. Randomization

We used Excel software with the “RANDOM.ENTRE(1;2)” formula. Number 1 was assigned to the control group, and number 2 to the intervention group. Each participant on the spreadsheet was randomly assigned one number.

### 2.4. Intervention

The intervention consisted of a 12-week course of in-person and in-nature exercise sessions. Two sessions were undertaken each week. One included walking in the mountains for 1 h and the other was a 1 h strength and resistance training session in an exercise park near the beach or outdoors on the university campus. There were 24 sessions in total. The sessions were led by one of the researchers, who is also a professor of sports physiology. The control group (CG) received the same information as the intervention group on the day of the baseline measurement about the importance of exercise and the recommended number of steps per day for health.

### 2.5. Study Variables and Data Collection

Data collection was performed through an online questionnaire using Google Forms. The baseline measurement was performed in March 2025 and the second measurement one day after the 12-week intervention. The questionnaire included the following variables: socio-demographic variables (age, sex, marital status, educational level, whether they had children), occupational variables (working and studying at the same time, number of hours working per week, engagement with university) and psychological variables (burnout, anxiety, stress, and depression) and mean number of steps per week (participants used smartphone-recorded step-counts). The independent variable was the exercise intervention.

The validated instruments for psychological variables were the Maslach Burnout Inventory—Student Survey (MBI-SS) for burnout (15 items covering emotional exhaustion, cynicism, and academic efficacy), the 10-item Perceived Stress Scale (PSS) for stress, the Utrecht Work Engagement Scale (UWES) for assessing work engagement (9-item short version (UWES-9)), and the 14-item Hospital Anxiety and Depression Scale (HADS) for measuring anxiety and depression.

### 2.6. Blinding

The intervention was not blinded, neither for the researchers nor the participants, due to its own characteristics. Data analysis was blinded for the researcher who performed the analysis.

### 2.7. Statistical and Qualitative Data Analysis

First, a descriptive analysis with central-tendency measures (mean and standard deviation) for continuous variables and a frequency analysis for categorical variables were performed.

The Kolmogorov–Smirnov test was used to check the normality of continuous variables. Baseline and post-intervention inter-group mean differences were calculated using the unpaired-sample Student’s *t*-test for continuous variables and the Chi-squared test for categorical variables.

Analyses were performed using the SPSS 28 statistical package.

### 2.8. Ethical Aspects

Before agreeing to participate, all participants received information about this study and were informed that they could leave this study at any time without having to give any reason. Participation was voluntary and anonymous. This study was approved by the ethics committee of the University of Granada (4638/CEIH/2024) in November of 2024.

## 3. Results

### 3.1. Baseline Descriptive Analysis of the Sample

**Figure 1:**
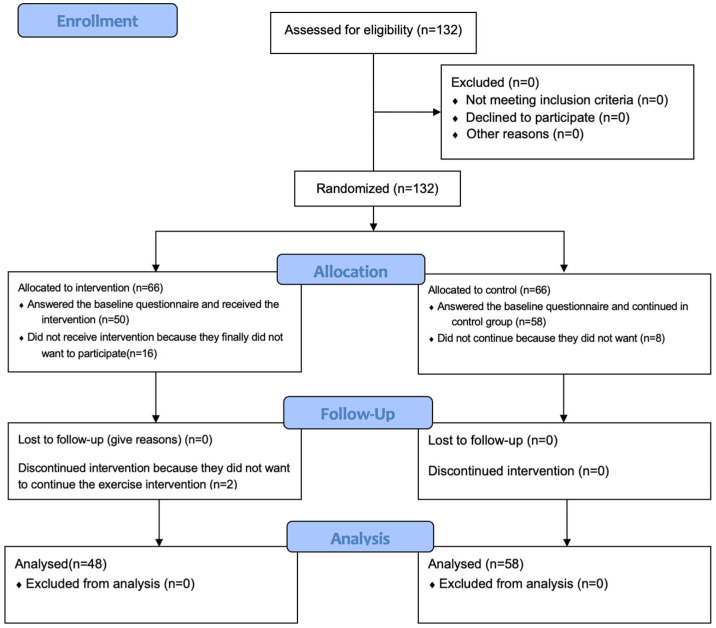
Flow diagram.

A total of 132 people were randomized and invited to participate. The final sample was n = 108 nursing students with n = 58 in the control group and n = 50 at baseline in the intervention group (81.81% response rate). Two people stopped participating during the intervention in the IG because they indicated they did not have time for the sports sessions. The flow diagram is shown in Figure 1.

Most of the participants were women (79.93%), were single (92.41%), and did not have children (96.02%). After comparing both groups at baseline, no differences were found between the intervention and the control group except regarding the variable “marital status”.

Table 1 shows the characteristics of each group at baseline and the results of the statistical tests for comparison between groups.

#### Post-Intervention Scores

During the 12-week intervention, two people did not participate in the initial measurement in the IG because they indicated they did not have time for the sports sessions, leaving n = 48. After the intervention, three variables were found to have significant differences between the CG and IG: emotional exhaustion (*p* = 0.028), stress (*p* = 0.037), and steps per day (*p* < 0.001). The mean values were lower in the IG than the CG: emotional exhaustion (3.28 points less), stress (2.85 points less), and steps per day (1381 steps more). The mean post-intervention values are shown in Table 2.

Furthermore, a 2 × 2 (group × time) repeated-measures ANOVA was performed. Table 3 shows the results of the 2 × 2 repeated-measures ANOVA. Emotional exhaustion (time and group effect), stress (group effect), depression (time effect), and average steps per day presented significant changes.

## 4. Discussion

We found that a scheduled sports intervention in nature for nursing students significantly reduced academic burnout, especially levels of emotional exhaustion and stress, compared to the control group.

These data are consistent with the scientific literature on the subject, which highlights the therapeutic role of outdoor physical exercise as a modulator of psychological well-being in adolescents, university students, and healthcare professionals in general [19,20].

After 12 weeks of outdoor aerobic and strength training, there was a significant reduction in emotional exhaustion and stress levels compared to the control group. This result is consistent with the study conducted by Ricardo-Rosales et al. [21], in which university students who underwent an aerobic exercise program reduced their level of emotional exhaustion by 26.4%, while the group that underwent an intervention based on strength training improved their emotional exhaustion to a lesser extent (19.5%).

Regarding the dimensions of burnout, in respect of depersonalization and efficacy, no statistically significant relationship was found with physical exercise in nature. Other studies have found a significant association between student participation in a sports program based on strength exercises and improved levels of depersonalization and efficacy [21,22]. This finding could be interpreted in terms of the 12-week sports program that our students followed, which is considered insufficient in duration to influence the dimensions of burnout syndrome mentioned above [22]. Similarly, it is possible that the physical exercise performed by the students was not of the intensity required for them to experience significant changes in their mental health and sleep quality, improved engagement, or reduced physical vulnerability to stress [23]. The lack of significant results in respect of cynicism and efficacy could be due, in addition to the duration of the program, to the sensitivity of the MBI-SS. Although widely validated, it has a greater capacity to detect changes in emotional exhaustion, while cynicism and academic efficacy usually require longer intervention periods or specific instruments with greater sensitivity to capture slight variations [24,25]. Some studies show that interventions tend to result in improvements mainly in emotional exhaustion, while the other dimensions require a longer exposure time [26].

Likewise, scientific literature has described the existence of several variables that act as mediators between physical exercise and the development of academic burnout, such as resilience and self-efficacy [21,27]. These mediators act to protect students from the psychological effects of stress experienced in the university environment [28].

Similarly, the benefits of practicing sports in nature for students’ mental health, especially in terms of anxiety control, stress management, and fatigue, have been widely described in the scientific literature [29,30]. Studies describe how nursing students show greater interest in caring for their physical and mental well-being as they progress through their studies [30,31,32]. In other words, students who are closer to practicing as nurses tend to sign up for more sports activities and create healthy lifestyle habits, especially after the lockdown due to the COVID-19 pandemic [31]. These data are consistent with the results obtained in this study, showing that students in the intervention group had a significantly higher average number of steps per day than those in the control group.

### 4.1. Limitations of This Study and Future Research

The main limitations of this study were as follows: randomization was performed using Excel, which, while allowing for accurate participant allocation, may be considered a weakness compared to software programs such as SPSS or R; the estimated sample size was not reached, which may have slightly influenced statistical power and the generalization of the results; due to the nature of the intervention, blinding was not possible for either participants or researchers, which may have introduced the risk of bias. However, to mitigate this risk, the statistical analysis was performed blindly and independently. Other potential limitations were the self-reporting of measurements and the possible influence of the proximity of the exam period on the students’ responses. These could have influenced the fact that the relationship between variables did not become significant in some analyses. Another limitation is the significant difference in marital status at baseline. Since this variable can influence social support and coping strategies, some effect on the results should be interpreted with caution. The findings should be interpreted with caution because non-probability convenience sampling was employed, including all second-year nursing students.

For future research, a longitudinal study could be conducted to collect data on the students who participated in this study and analyze whether practicing outdoor sports during their formative years has had a significant influence on the development of burnout in the workplace. Further, future research should consider variables such as resilience or self-efficacy because they can influence burnout and have not been included in this study.

### 4.2. Strengths of This Study

This study presents results that support the robustness of its conclusions. The use of a randomized clinical trial design provided methodological rigor and allowed a reliable comparison between intervention and control groups. In addition, the application of validated measurement instruments, including MBI-SS, the PSS, and the HADS, ensured accuracy and facilitated comparability with previous research. Another strength lies in the relevance of the topic, as burnout among nursing students is a pressing issue with significant implications for both academic performance and future professional practice.

This trial is the first, to our knowledge, to evaluate the effectiveness of physical activity in nature on academic burnout in nursing students. Unlike other studies that have focused on general exercise or a nonspecific population, our work focuses on a particularly vulnerable group exposed to high academic and clinical demands. These types of interventions are relevant for nurses because they promote mental health, resilience, and healthy lifestyle habits during the university years, factors that may influence the well-being of professionals in the future and, indirectly, the quality of care.

## 5. Conclusions

The findings of this study suggest that including a nature-based physical exercise schedule may be an effective strategy for preventing academic burnout in each year of the nursing degree program. These organized outdoor activities appear to be effective in improving the levels of emotional fatigue and stress in nursing students, as well as increasing the average number of steps per week.

## Figures and Tables

**Table 1:** Baseline characteristics. n = 108 (control group (CG) n = 58; intervention group (IG) = 50).

Variable	Mean (Standard Deviation)	*t*	*p*-Value	
Age	CG: 22.28 (7.87) IG: 21.24 (1.83)	12.951	0.332	
Working hours per week	CG: 3.7 (12.22) IG: 3.65 (12.22)	0.311	0.982	
Mean steps per day	CG: 7912.04 (1150.88) IG: 8237.64 (1284.77)	0.986	0.171	
Emotional exhaustion	CG: 14.86 (7.76) IG: 12.44 (7.28)	0.259	0.083	
Cynicism	CG: 4.06 (4.13) IG: 4.55 (5.08)	1.151	0.587	
Academic efficacy	CG: 26.54 (5.42) IG: 23.68 (9.20)	16.381	0.057	
Stress	CG: 18.76 (6.75) IG: 18.44 (7.08)	0.024	0.816	
Anxiety	CG: 9.46 (3.42) IG: 9.28 (3.79)	0.820	0.793	
Depression	CG: 12.28 (1.96) IG: 12.65 (2.54)	2.401	0.063	
Engagement	CG: 3.96 (1.18) IG: 3.50 (1.54)	2.912	0.073	
**Categorical variables**	**Group**	**Categories %**	**Chi-squared**	***p***
Sex	CG	Male: 16% Female: 84%	1.09	0.295
	IG	Male: 24.4% Female: 75.86%		
Marital Status	CG	Single: 90% Married: 10%	96.70	<0.001
	IG	Single: 94.82% Married: 5.18%		
Children	CG	No: 94%	3.57	0.167
	IG	No: 98.04%		
Working and studying	CG	Yes: 12%	1.46	0.227
	IG	Yes: 20.64%		
Emotional exhaustion	CG	Low (50%), medium (16%), high (34%)	2.66	0.264
	IG	Low (63.64%), medium (15.48%), high (20.64%)		
Cynicism	CG	Low (74%), medium (22%), high (4%%)	0.448	0.799
	IG	Low (70.52%), medium (22.36%), high (6.88%)		
Academic efficacy	CG	High (44%), medium (34%), low (22%)	2.76	0.252
	IG	High (43%), medium (22.36%), low (34.4%)		
Burnout	CG	46%	3.72	0.444
	IG	37.84%		

Note: control group = CG; intervention group 1 = IG1; gamified intervention group 2 = IG2.

**Table 2:** Post-intervention means (GC n = 58; GI n = 48) and comparation between groups.

Variable	Mean (Standard Deviation)	*t*	*Cohen’s D (95%CI)*	*p-Value*
Emotional exhaustion	GC: 17.24 (7.31) IG: 13.96 (6.39)	2.23	0.483 (0.05, 0.91)	0.028
Cynicism	CG:5.29 (5.43)	−0.28	−0.061 (−0.48, 0.36)	0.779
	IG: 5.60 (4.84)			
Professional efficiency	CG: 26.05 (4.69)	0.699	0.151 (−0.27, 0.57)	0.486
	IG: 25.15 (6.69)			
Stress	CG: 20.35 (7.25) IG: 17.50 (5.36)	2.11	0.456 (0.02, 0.88)	0.037
Anxiety	CG: 9.59 (2.76) IG: 9.28 (3.79)	0.46	0.101 (0.10, −0.32)	0.641
Depression	CG: 11.97 (2.51) IG: 11.39 (2.87)	1.06	0.229 (0.22, −0.19)	0.291
Engagement	CG: 4.04 (1.04) IG: 4.06 (1.13)	−0.10	−0.22 (−0.44, 0.40)	0.920
Average steps per day	CG: 8573 (1337.22) IG: 9954.55 (1204.85)	−5.06	−1.09 (−1.54, −0.63)	<0.001

Note: CI = confidence interval; CG = control group; IG = intervention group (sports intervention).

**Table 3:** Repeated-measures ANOVA for pre-post measures.

Variable	Mean (Standard Deviation)	Effect	F	Partial η^2^	*p*-*Value*
Emotional exhaustion	CG pre: 14.86 (7.76) CG post: 17.24 (7.31) IG pre: 12.44 (7.28) IG post: 13.96 (6.39)	Time	5.85	0.056	0.017
		Group	16.28	0.141	<0.001
		Time * group	0.002	0.00	0.967
Cynicism	CG pre: 4.06 (4.13) CG post: 5.29 (5.43) IG pre: 4.55 (5.08) IG post: 5.60 (4.84)	Time	3.36	0.031	0.070
		Group	0.34	0.003	0.561
		Time * group	0.02	0.000	0.881
Professional efficiency	CG pre: 26.54 (5.42) CG post: 26.05 (4.69) IG pre: 23.68 (9.20) IG post: 25.15 (6.69)	Time	1.09	0.013	0.3
		Group	2.37	0.027	0.128
		Time * group	0.66	0.008	0.42
Stress	CG pre: 18.76 (6.75) CG post: 20.35 (7.25) IG pre: 18.44 (7.08) IG post: 17.50 (5.36)	Time	0.04	0.000	0.852
		Group	5.31	0.058	0.024
		Time * group	0.45	0.005	0.505
Anxiety	CG pre: 9.46 (3.42) CG post: 9.59 (2.76) IG pre: 9.28 (3.79) IG post: 9.27 (3.36)	Time	0.00	0.000	0.985
		Group	1.03	0.016	0.314
		Time * group	0.68	0.011	0.414
Depression	CG pre: 12.28 (1.96) CG post: 11.97 (2.51) IG pre: 12.65 (2.54) IG post: 11.39 (2.87)	Time	4.98	0.055	0.028
		Group	0.30	0.003	0.584
		Time * group	1.47	0.017	0.228
Engagement	CG pre: 3.96 (1.18) CG post: 4.04 (1.04) IG pre: 3.50 (1.54) IG post: 4.06 (1.13)	Time	3.34	0.037	0.071
		Group	0.70	0.008	0.406
		Time * group	0.86	0.010	0.355
Average steps per day	CG pre: 7912.04 (1150.88) CG post: 8573 (1337.22) IG pre: 8237.64 (1284.77) IG post: 9954.55 (1204.85)	Time	45.32	0.304	<0.001
		Group	28.18	0.213	<0.001
		Time * group	9.41	0.083	0.003

Note: CG = control group; IG = intervention group (sports intervention); * = interacion between time and group.

## Data Availability

The raw data supporting the conclusions of this article will be made available by the authors on request.

## Supplementary Materials

The following supporting information can be downloaded at: https://www.mdpi.com/article/10.3390/healthcare13192510/s1. CONSORT checklist [33].

## Abbreviations

The following abbreviations are used in this manuscript:

CG	Control group
IG	Intervention group
